# A diamond layer in Mercury’s deep interior

**DOI:** 10.1038/s41467-024-49497-2

**Published:** 2024-06-14

**Authors:** Megan D. Mouser

**Affiliations:** Jacobs Technology, NASA Johnson Space Center, Houston, TX USA

**Keywords:** Planetary science, Geochemistry, Core processes

## Abstract

Surface observations suggest that carbon may exist in Mercury’s interior. Under planetary mantle pressures and temperatures, carbon may be present as a stable diamond layer which may have important implications for the physical and chemical properties of Mercury.

Mercury is an unusual planet in our Solar System. Like Earth, it has an exosphere^1^ and a magnetic field^2^, but it has unique traits that make it an outlier from the other rocky bodies in our Solar System, such as its large core-to-mantle ratio^3^ and exotic surface chemistry^4,5^. Despite their differences, the magma ocean hypothesis suggests that early in the formation of terrestrial planets—Earth, Moon, Venus, Mars, and Mercury—they all went through a large global melting stage that led to the segregation of a metal core from the silicate magma, which then cooled and crystallized into the mantle and crust (Fig. 1). In their recent work^6^, Xu and colleagues suggest that during these early stages of Mercury’s formation, there may have been a diamond layer at the core–mantle boundary according to a combination of laboratory experiments and modeling approaches that explore carbon phase stability under Mercurian conditions.

**Fig. 1:**
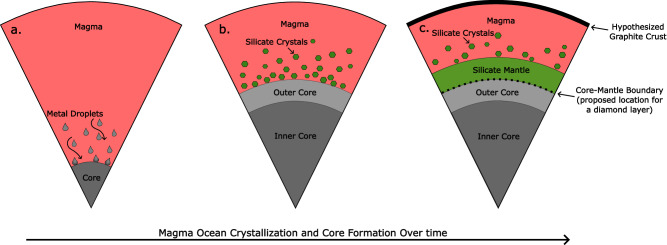
The evolution of Mercury’s magma ocean. Xu and colleagues^6^ propose the existence of a diamond layer at the core–mantle boundary in Mercury, which formed during the process of magma ocean crystallization and from post-magma ocean crystallization percolation from the core. **a** Metal begins to precipitate from the magma ocean and sequester into the core. **b** Core has formed, and silicate crystals are starting to form as the magma cools. **c** Advanced crystallization of the magma ocean and formation of the mantle.

## An overview of Mercury

Our knowledge of Mercury comes from ground-based techniques^7^, such as radio telescopes, and orbital data^4,8–10^, largely from NASA orbital missions: Mariner 10 (1973–1975) and MESSENGER (Mercury Surface, Space Environment, Geochemistry and Ranging; 2004–2014). More data are to come from the upcoming ESA-JAXA mission BepiColombo, which is set to orbit Mercury in late 2025 and has already provided powerful images and analysis from Mercury flybys.

Data from these missions indicate a unique Mercurian chemistry. Its surface is highly reduced^5^ with low levels of iron (<3 wt%^8^), relatively high sulfur (>4 wt%^9^), and regions of low reflectance, interpreted as carbon-rich regions^10^. These observations have prompted research into Mercury’s bulk composition and questions as to how it evolved to produce such a unique surface with distinct volcanic terranes. Like the other terrestrial planets in our Solar System, it is suggested to have started with the magma ocean stage^11–14^. In order to understand the mineralogy and structure of planetary mantles during and after the magma ocean stage, we rely on laboratory experiments which explore the melting and crystallization of planetary compositions at a range of high-pressures and high-temperatures, as well as geophysical modeling that considers current measurements and predictions of planetary properties such as gravity, mass, density, and magnetic fields.

## Carbon in Mercury’s crystallizing magma ocean

With carbon observed in the low reflectance regions on the surface of Mercury, and potentially present in the interior, Xu et al.^6^ explore carbon phase stability and crystallization in the magma ocean scenario. Owing to similarities between bulk chemistry, sulfide content, and reduced oxidation state, enstatite chondrites are considered analogs to Mercury’s bulk composition. Xu and colleagues therefore used modified enstatite chondrite compositions as the starting material for a series of high-pressure and high-temperature experiments that are designed to examine the phases present at deep mantle conditions at pressures of 7 GPa and temperatures up to 2213 K. They identified silicate minerals olivine, orthopyroxene, clinopyroxene, and garnet along with FeS and MgCaFeS sulfides, which align with observations from previous work^11,13,14^, and provide important constraints on the major minerals at these deep mantle conditions.

In order to understand carbon phase stability and crystallization under the magma ocean scenario, Xu et al.^6^ then combined their experimental results with a model to predict the graphite-diamond transition. They found that if sulfur is absent, graphite is stable, but if sulfur is present, diamond may be stable under the correct conditions. As observed by low reflectance regions on Mercury’s surface, graphite would be the dominate phase in the shallower portions of the mantle and crust, while any diamond growth would have been limited to deeper parts of the core and mantle. In theory, under relevant pressure, temperature, oxidation state, and carbon solubility conditions, calculations suggest that a diamond layer of between 0.1 and 200 m could theoretically be possible at the core-mantle boundary as a product of magma ocean crystallization.

This layer of diamond could form in different ways. First, it could be crystallization at high pressures from the carbon-saturated magma ocean. Second, possibly as a trapped graphite transformation in a cooling Mercurian interior. And finally, from carbon in Mercury’s core, crystallizing to diamond and percolating to the core–mantle boundary, contributing to the formation of a late diamond layer. The authors conclude that diamond mobilizing from the core, in addition to some crystallization from the magma ocean, is the most likely scenario that may produce a significant enough diamond layer.

## Implications of diamond in Mercury

A planet’s moment of inertia (MOI) characterizes the mass distribution within its interior and can provide insight into size and density estimates of the core and mantle. A recently determined MOI^15^ could indicate a deeper core–mantle boundary than previously suggested, which may change the stability of silicate minerals and carbon phases present deeper in the mantle. In an effort to understand the lower MOI value, Xu et al.^6^ explore the possibility of carbon’s effect in the deep mantle and find that a diamond-bearing layer of approximately 10–15 km, a thicker layer than estimated in their previous crystallization model, could satisfy the updated MOI.

The diamond layer, whether 0.1 or 15 km thick, would be conductive. As it sits atop the liquid outer core, this conductivity may assist in the generation of Mercury’s magnetic field.

Finally, carbon is one of the volatile elements crucial for understanding planetary formation, including the generation of atmospheres and habitable planets. This work helps to constrain phase stability of carbon in Mercury and, therefore, impacts understanding of volatile element storage in planetary interiors more generally, which may help to constrain the evolution of other carbon-rich bodies, even exoplanets.

## The future of Mercury science

Past missions, in particular MESSENGER, provided a wealth of information on Mercury. Results from the BepiColombo mission will add to this knowledge, offering a more detailed insight into the Solar System’s inner-most planet; a worthy goal in itself. From this understanding of Mercury, we will also learn more about the formation of terrestrial planets in general, from Earth and our neighbors, to distant exoplanets.
